# Genome-wide analysis of LTR-retrotransposon diversity and its impact on the evolution of the genus *Helianthus* (L.)

**DOI:** 10.1186/s12864-017-4050-6

**Published:** 2017-08-18

**Authors:** Flavia Mascagni, Tommaso Giordani, Marilena Ceccarelli, Andrea Cavallini, Lucia Natali

**Affiliations:** 1 Department of Agriculture, Food and Environment, University of Pisa, 56124 Pisa, Italy; 2 Department of Chemistry, Biology and Biotechnology, University of Perugia, 06123 Perugia, Italy

**Keywords:** Annual and perennial habit, Plant genome evolution, *Helianthus*, LTR-retrotransposons, Repetitive DNA, Comparative genomics

## Abstract

**Background:**

Genome divergence by mobile elements activity and recombination is a continuous process that plays a key role in the evolution of species. Nevertheless, knowledge on retrotransposon-related variability among species belonging to the same genus is still limited. Considering the importance of the genus *Helianthus*, a model system for studying the ecological genetics of speciation and adaptation, we performed a comparative analysis of the repetitive genome fraction across ten species and one subspecies of sunflower, focusing on long terminal repeat retrotransposons at superfamily, lineage and sublineage levels.

**Results:**

After determining the relative genome size of each species, genomic DNA was isolated and subjected to Illumina sequencing. Then, different assembling and clustering approaches allowed exploring the repetitive component of all genomes. On average, repetitive DNA in *Helianthus* species represented more than 75% of the genome, being composed mostly by long terminal repeat retrotransposons. Also, the prevalence of *Gypsy* over *Copia* superfamily was observed and, among lineages, *Chromovirus* was by far the most represented. Although nearly all the same sublineages are present in all species, we found considerable variability in the abundance of diverse retrotransposon lineages and sublineages, especially between annual and perennial species.

**Conclusions:**

This large variability should indicate that different events of amplification or loss related to these elements occurred following species separation and should have been involved in species differentiation. Our data allowed us inferring on the extent of interspecific repetitive DNA variation related to LTR-RE abundance, investigating the relationship between changes of LTR-RE abundance and the evolution of the genus, and determining the degree of coevolution of different LTR-RE lineages or sublineages between and within species. Moreover, the data suggested that LTR-RE abundance in a species was affected by the annual or perennial habit of that species.

**Electronic supplementary material:**

The online version of this article (doi:10.1186/s12864-017-4050-6) contains supplementary material, which is available to authorized users.

## Background

Transposable elements (TEs) play a key role in the evolution of species [1]. They can drive rapid genome remodelling by creating chromosomal rearrangements and new regulatory gene networks, acting as an endogenous force that promotes reproductive isolation [2]. Moreover, the mutagenic action of TEs creates substantial genetic variability [3], novel functions by fine-tuning gene activity [4, 5], and they are also a major driver of genome size evolution [6–8].

Although many kinds of TEs have been identified, they generally fall into two major classes based on their transposition mechanisms [9]. Retrotransposons, or Class I elements, transpose by an RNA intermediate which is reverse transcribed from the genomic copy and integrated in a new position elsewhere in the genome [10]. DNA transposons, or Class II elements, can move using an enzymatic method for excision from the chromosome and reintegration in a new location [9].

Retrotransposons (REs) are the most common class of elements, making up the bulk of many genomes [2, 11]. They can be classified into five taxonomic orders [9], among which long terminal repeat (LTR) REs and non-LTR-REs differ in the mechanism of integration. The size of LTR-REs varies from a few hundred base pairs to over 10 kb, in which two identical LTRs side a region containing open reading frames (ORFs) for Gag and for Pol. Pol encodes a polyprotein with protease, reverse transcriptase (RT), RNaseH, and integrase enzyme domains, which are necessary for the replication and the integration of the elements in the host chromosomes [10].

LTR-REs are the most abundant order in plants, especially those belonging to the *Copia* and *Gypsy* superfamilies [9] which differ in the position of the integrase domain within the ORFs [10]. *Copia* and *Gypsy* superfamilies can be subdivided into several major evolutionary lineages [12, 13]. The main *Copia* lineages are: *AleI/Retrofit/Hopscotch*, *AleII*, *Angela*, *Bianca*, *Ivana/Oryco*, *TAR/Tork*, and *Maximus/SIRE* [12]. On the other hand, the most frequent *Gypsy* lineages are: *Ogre/Tat* [14], *Athila* [15], and *Chromovirus*, a lineage which is especially abundant in centromeric regions and carries a chromodomain at the 5′-end of the coding portion [13, 16]. In certain species, four sublineages (*Galadriel*, *Tekay*, *CR*, and *Reina*) of *Chromoviruses* have been distinguished according to the positions of the chromodomain and the polypurine tract, and to the LTR length [17].

Studies on the impact of RE proliferation and loss on genome structure and evolution of plant species have been performed especially in species with small- or medium-sized genomes. In angiosperms, large sized genomes have been studied especially in monocotyledonous species such as maize (2.3 gigabase pairs, [18]) and barley (5.1 gigabase pairs, [19]). For this reason, we decided to study genome size, structure and evolution in a dicotyledonous genus with a large genome, such as *Helianthus* (for example, *H. annuus* has a genome size of 3.3 Gbp, [20]).

This genus, which belongs to the Asteraceae family, includes 49 outcrossing species from different habitats and with a remarkable level of variability [21], including differences in phenotypic traits such as reproductive timing, branching patterns, height [22, 23] and especially habitat preferences. The study of Timme et al. [24] provided evidence for multiple, independent hybrid speciation and/or polyploidy events subdividing sunflowers into four different sections: a monophyletic annual section *Helianthus*, a polyphyletic section *Ciliares* and the monotypic section *Agrestis*, all of which were encompassed by a large polyphyletic section, *Divaricati*.

It has been evident for more than a decade that the sunflower (*H. annuus*) genome contains many thousands of TEs [25–29]. In particular, the repetitive fraction of the sunflower genome contains more than 81% TEs, 77% of which are LTR-REs [29]. Among LTR-REs, elements belonging to the *Gypsy* superfamily are 2.3 times more represented than those belonging to the *Copia* superfamily [27]. Furthermore, massive transposition of *Gypsy*-like LTR-REs is supposed to have driven a rapid speciation (in less than 60 generations in one case) of three species of the *Helianthus* section (*H. anomalus, H. deserticola* and *H. paradoxus*), derived from the same two parental species (*H. annuus* and *H. petiolaris*). The genomes of these hybrid taxa are 50% larger than the genome of parental lines as the result of bursts of transposition. Further analyses of these *Helianthus* species have shown that RE proliferation has occurred even in relatively recent events [30].

Although many data are available on *Helianthus* evolution, massively parallel sequencing techniques are providing new possibilities to investigate genome structure and its impact on speciation. The use of these technologies within a computational framework led to the identification of a so called “metagenome” of repetitive elements of the species analysed, allowing us to address many facets of the dynamics of changes of the genomic repetitive component within the largely unexplored genus *Helianthus*. These include: i) establishing the extent of intrageneric repetitive DNA variation, especially considering LTR-REs, at superfamily, lineage and sublineage levels; ii) studying the relationship between changes of LTR-RE abundance and the evolution of a genus; iii) investigating whether there is a relation between annual or perennial habits of species and LTR-RE abundance; iv) checking whether different LTR-RE lineages or sublineages have coevolved; v) studying variations in the dynamics (amplification, loss, proliferation dating) of specific LTR-REs among species.

## Results

### Genome characterization of *Helianthus* species

In order to classify repetitive sequences and identify their homologous groups in individual genomes, 10 species and one subspecies out of 49 *Helianthus* species were selected (see Table 1). Of these, two species belong to the section *Helianthus* (*H. annuus* and *H. petiolaris*, with two subspecies: *H. petiolaris* ssp. *petiolaris*, and *H. petiolaris* ssp. *fallax*), one represents the monotypic annual section *Agrestis* (*H. agrestis*), another annual species (*H. porteri*) and six perennial species belong to the section *Divaricati* (according to [24]). Concerning the six perennial species of the section *Divaricati*, two diploid species (*H. divaricatus* and *H. giganteus*), three tetraploid species (*H. hirsutus*, *H. californicus* and *H. laevigatus*) and one hexaploid species (*H. tuberosus*) were selected.

**Table 1:** *Helianthus* species analysed and number of Illumina reads used for the analyses

Species name	Section	*Habits*	Acronym	ARS-GRIN Id Code ^a^	Raw reads	Trimmed reads (90 bp)
*H. agrestis*	*Agrestis*	annual	AGR	Ames 30845	40,986,566	40,808,492
*H. annuus*	*Helianthus*	annual	ANN	PI 435540	18,577,580	17,366,768
*H. petiolaris* ssp. *fallax*	*Helianthus*	annual	PFA	PI 435805	15,969,364	14,881,524
*H. petiolaris* ssp. *petiolaris*	*Helianthus*	annual	PPE	PI 435799	14,086,950	13,911,650
*H. porteri*	*Divaricati*	annual	POR	PI 649911	14,436,386	14,321,186
*H. divaricatus*	*Divaricati*	perennial	DIV	PI 435675	25,520,422	25,318,342
*H. giganteus*	*Divaricati*	perennial	GIG	Ames 21040	15,299,732	14,925,322
*H. californicus*	*Divaricati*	perennial	CAL	PI 649941	50,810,594	50,231,784
*H. hirsutus*	*Divaricati*	perennial	HIR	PI 547203	43,742,044	42,909,176
*H laevigatus*	*Divaricati*	perennial	LAE	PI 503227	36,682,094	36,393,234
*H. tuberosus*	*Divaricati*	perennial	TUB	Ames 22229	52,488,912	51,451,774

^a^germplasm information for each accession can be accessed at https://npgsweb.ars-grin.gov/gringlobal/search.aspx

To achieve our task, we sequenced genomic DNA from one individual for each species, treating it as a “type” representative of the species. Concerning *H. annuus*, previous studies documented high variability of the repetitive component between wild and cultivated genotypes [31]. Since the present analysis has focused on wild species of the genus *Helianthus*, a wild accession from Illinois was chosen to represent *H. annuus*; this particular accession exhibits average features among wild *H. annuus* genotypes [31].

Since genome size was not available for some species (*H. porteri*, *H. californicus*, *H. hirsutus* and *H. laevigatus*), we measured genome size of all species to obtain comparable values. Genome size was evaluated cytophotometrically, measuring the absorption of prophase nuclei (which have a 4C-DNA content) of root apices after Feulgen staining. Moreover, for one species, chromosome number was not precisely ascertained. Since rare diploid forms were reported for tetraploid *H. hirsutus* [32], we decided to check the chromosome number of our materials. All chromosome numbers were in agreement with previous works (reviewed in [32]). The *H. hirsutus* accession used in our experiments resulted tetraploid, as expected. Table 2 reports the chromosome number and the relative genome size of the analysed species.

**Table 2:** *Helianthus* analyzed species and subspecies, their chromosome number and 4C-Feulgen DNA absorption. Three seedlings per species were analysed

Species name	Section	Chromosome number	4C-Feulgen DNA absorption (± SE)
*H. agrestis*	*Agrestis*	34	64.963 ± 0.306
*H. annuus*	*Helianthus*	34	27.865 ± 0.099
*H. petiolaris* ssp. *fallax*	*Helianthus*	34	23.181 ± 0.141
*H. petiolaris* ssp. *petiolaris*	*Helianthus*	34	26.151 ± 0.097
*H. porteri*	*Divaricati*	34	28.964 ± 0.218
*H. divaricatus*	*Divaricati*	34	32.828 ± 0.141
*H. giganteus*	*Divaricati*	34	34.246 ± 0.183
*H. californicus*	*Divaricati*	68	71.352 ± 0.442
*H. hirsutus*	*Divaricati*	68	61.023 ± 0.233
*H. laevigatus*	*Divaricati*	68	86.956 ± 0.377
*H. tuberosus*	*Divaricati*	102	67.811 ± 0.397

Interestingly, diploid *H. agrestis* showed a 4C-DNA absorption almost two times higher than the largest value measured for a diploid species (*H. giganteus*), similarly to what has been previously reported [33]. Note that the tetraploid species *H. californicus* and *H. laevigatus* had larger genome sizes than expected, based on genome sizes of diploid species (ranging from 23.2 to 34.2 arbitrary units). On the contrary, the hexaploid *H. tuberosus* had a 4C-value smaller than expected, in agreement with other data in the literature [34, 35].

The repetitive component of each species was then characterized by applying the RepeatExplorer pipeline [36], using a number of reads proportional to the ploidy level of each species.

In Table 3, we report the number of clustered sequence reads, i.e. the repetitive DNA, for each species and their corresponding percentages within genomes (ranging from 73.6 to 84.2%). *Helianthus agrestis* and *H. porteri* showed the highest percentage of repeated sequences, *H. petiolaris* spp. *fallax* and *H. californicus* the lowest.

**Table 3:** *Helianthus* analyzed species and subspecies, their ploidy level and number of reads analyzed by RepeatExplorer

Species name	Section	Ploidy level	Reads used for the comparative analysis	Total reads processed by RepeatExplorer	Reads in clusters	Reads in singlets
*H. agrestis*	*Agrestis*	2×	1000,000	259,362	218,464 (84.2%)	40,898 (15.8%)
*H. annuus*	*Helianthus*	2×	1000,000	260,544	196,995 (75.6%)	63,549 (24.4%)
*H. petiolaris* ssp. *fallax*	*Helianthus*	2×	1000,000	260,192	191,401 (73.6%)	68,791 (26.4%)
*H. petiolaris* ssp. *petiolaris*	*Helianthus*	2×	1000,000	259,278	198,005 (76.4%)	61,273 (23.6%)
*H. porteri*	*Divaricati*	2×	1000,000	260,872	207,361 (79.5%)	53,511 (20.5%)
*H. divaricatus*	*Divaricati*	2×	1000,000	259,756	200,072 (77.0%)	59,684 (23.0%)
*H. giganteus*	*Divaricati*	2×	1000,000	261,508	204,244 (78.1%)	57,264 (21.9%)
*H. californicus*	*Divaricati*	4×	2,000,000	520,754	386,259 (74.2%)	134,495 (25.8%)
*H. hirsutus*	*Divaricati*	4×	2,000,000	519,636	396,207 (76.2%)	123,429 (23.8%)
*H. laevigatus*	*Divaricati*	4×	2,000,000	521,176	406,256 (77.9%)	114,920 (22.1%)
*H. tuberosus*	*Divaricati*	6×	3,000,000	781,686	620,615 (79.4%)	161,071 (20.6%)

### Composition of *Helianthus* repetitive fraction

**Fig. 1:**
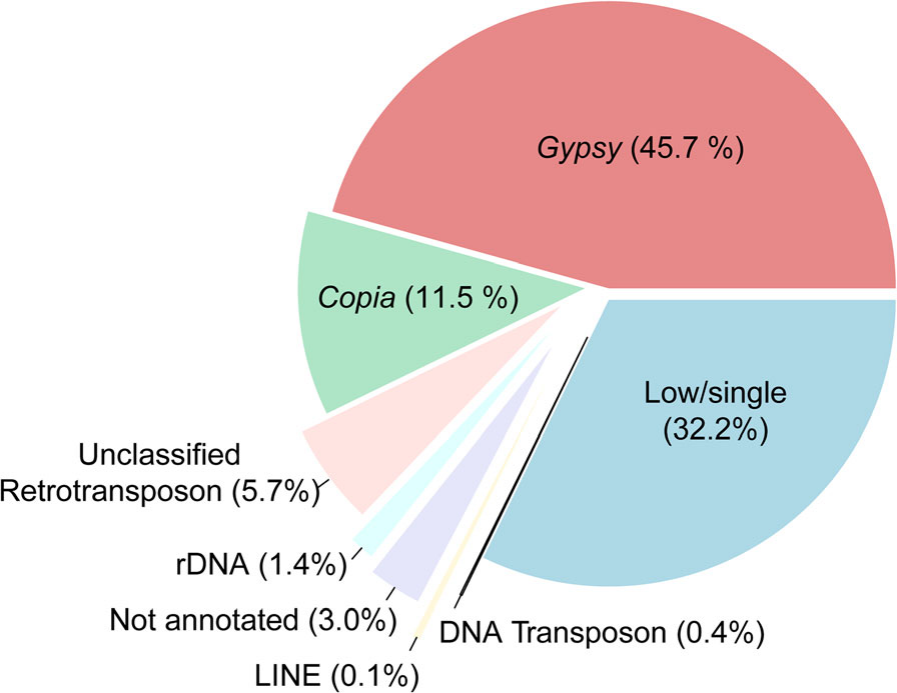
The repeat class distribution of the 338 top (most frequent) clusters obtained performing a hybrid clustering with a random set of reads of sunflowers (proportionally to species ploidy level) using RepeatExplorer. The percentage of reads included in repeat class is reported inside brackets

The “metagenome” structure of the analysed pool of species is reported in Fig. 1 and Table 4, based on the genomic proportion of the 338 hybrid clusters, each representing >0.01% of the analysed reads. The LTR-RE-related clusters composed the bulk of highly and moderately repeated sequences in the *Helianthus* genomes, as previously reported for *H. annuus* [27, 29]. The DNA transposons and non-LTR-REs were under-represented; unannotated repeats accounted for 3.0% of the genome. It is also presumable that other repeat remnants could be found among low-repeated/single sequences.

**Table 4:** Description of the 248 clusters obtained by hybrid clustering using RepeatExplorer and annotated as LTR-RE, and the percentage of analyzed reads per cluster

Superfamily	Lineage	Number of clusters	Reads in clusters [%]
*Copia*	*AleII*	4	0.84
	*Angela*	5	0.66
	*Bianca*	1	0.13
	*Maximus/SIRE*	32	6.46
	*TAR/Tork*	7	0.51
	Unknown	7	2.88
	Total	56	11.48
*Gypsy*	*Athila*	25	3.18
	*Chromovirus*	105	35.29
	*Ogre/Tat*	20	4.03
	Unknown	14	3.15
	Total	164	45.65
Unknown		28	5.7

Table 4 reports the classification of 248 clusters annotated as LTR-REs. We found 56 clusters for the *Copia* superfamily (11.48% of analysed reads) and 164 clusters for the *Gypsy* superfamily (45.65% of analysed reads).

Focusing on LTR-REs, these elements were characterized at the lineage level (Table 4): five lineages were identified among *Copia* retrotransposons (*AleII*, *Angela*, *Bianca*, *Maximus/SIRE*, and *TAR/Tork*) and three lineages were identified among *Gypsy* elements (*Chromovirus*, *Ogre/Tat*, and *Athila*).

The genome proportions of the different RE lineages in the selected species are reported in Table 5. Cluster-related repeats annotated as LTR-REs ranged from 58.36% of the genome in *H. petiolaris* ssp. *fallax* to 74.99% in *H. agrestis*. *Gypsy* LTR-REs ranged from 33.05% in *H. porteri* to 57.03% in *H. agrestis*, and they were overrepresented compared to *Copia* elements, whose percentages ranged between 8.31% in *H. divaricatus* and 22.79% in *H. porteri*.

**Table 5:** Genome proportion of LTR-RE sequences and maximum percentage of variation among the analyzed species and subspecies (acronyms as defined in Table 1)

Superfamily	Lineage	Genome proportion											Max. percentage of variation
		ANN	PFA	PPE	DIV	HIR	TUB	GIG	CAL	LAE	POR	AGR	
*Copia*	*AleII*	0.89	0.85	0.75	0.59	0.82	0.75	0.82	1.32	0.66	1.07	0.57	56.94
	*Angela*	0.79	0.82	0.59	0.57	0.90	0.71	0.93	0.46	0.67	0.45	0.29	69.15
	*Bianca*	0.19	0.21	0.11	0.14	0.18	0.12	0.17	0.07	0.11	0.11	0.03	85.60
	*Maximus/SIRE*	6.72	7.08	7.21	4.31	6.11	6.04	6.40	4.41	4.90	15.56	7.08	72.32
	*TAR/Tork*	0.41	0.49	0.58	0.59	0.48	0.52	0.47	0.32	0.62	0.63	0.62	48.22
	Unknown	3.43	2.93	2.83	2.11	3.09	2.84	3.02	2.76	2.28	4.97	2.04	58.92
	*Total*	12.44	12.39	12.08	8.31	11.58	10.98	11.81	9.34	9.23	22.79	10.62	63.53
*Gypsy*	*Athila*	2.81	3.00	3.88	3.29	2.64	3.29	2.51	2.87	3.65	4.07	3.19	38.47
	*Chromovirus*	30.82	29.92	31.52	37.85	34.10	36.87	36.73	33.83	37.02	25.25	52.18	51.62
	*Ogre/Tat*	5.96	3.98	4.64	3.76	4.85	5.22	4.42	2.83	3.93	1.78	1.02	82.85
	Unknown	2.75	3.13	4.11	4.25	2.85	3.50	2.36	3.91	3.59	1.95	0.64	85.05
	*Total*	42.34	40.04	44.14	49.15	44.44	48.88	46.02	43.44	48.18	33.05	57.03	42.04
LTR-RE unclassified		6.00	5.93	5.51	5.40	5.40	5.31	5.66	5.97	5.49	5.98	7.34	27.59
Annotated LTR-RE total		60.77	58.36	61.73	62.86	61.42	65.18	63.49	58.75	62.90	61.83	74.99	22.17
LTR-*Gypsy*/LTR-*Copia*		3.40	3.23	3.66	5.91	3.84	4.45	3.90	4.65	5.22	1.45	5.37	

The ratio between the genomic proportions of *Gypsy* and *Copia* elements differed among species, from 5.91 in *H. divaricatus* to 1.45 in *H. porteri*. Interestingly, species of the section *Helianthus* showed a ratio ranging from 3.23 to 3.66, i.e. lower than that of perennial species (from 3.84 in *H. hirsutus* to 5.91 in *H. divaricatus*); the monophyletic section *H. agrestis* and *H. porteri* showedthe extreme ratios (5.37 and 1.45, respectively). The *H. porteri* low ratio (1.45) is peculiar in the *Helianthus* genus, in which *Gypsy* elements are typically reported to be much more abundant than *Copia* ones.

The maximum percentage variation of genome proportion of each LTR-RE superfamily or lineage among the 10 species and one subspecies of sunflowers gave us an estimation of genome proportion variability of *Gypsy* and *Copia* elements within the genus *Helianthus*. Such variability was large for each superfamily, and it was especially larger for *Copia* (63.53%) than for *Gypsy* (42.04%) and unknown elements (27.59%).

Among *Copia* REs, only *Maximus/SIRE* elements showed an average genome proportion higher than 1%. In contrast, *Bianca* REs were barely represented. Each *Gypsy* lineage accounted on average for more than 3% of the genome, with *Chromoviruses* exceeding or being around 30% in each species, excluding *H. porteri* (25.25%); in *H. agrestis*, *Chromoviruses* accounted for about 50% of the genome.

The abundance of each cluster in the different *Helianthus* species as determined using RepeatExplorer was confirmed by mapping Illumina reads on the contigs belonging to each cluster and counting the number of mapped reads (Additional file 1: Figure S1).

### LTR-retrotransposons and *Helianthus* phylogeny

**Fig. 2:**
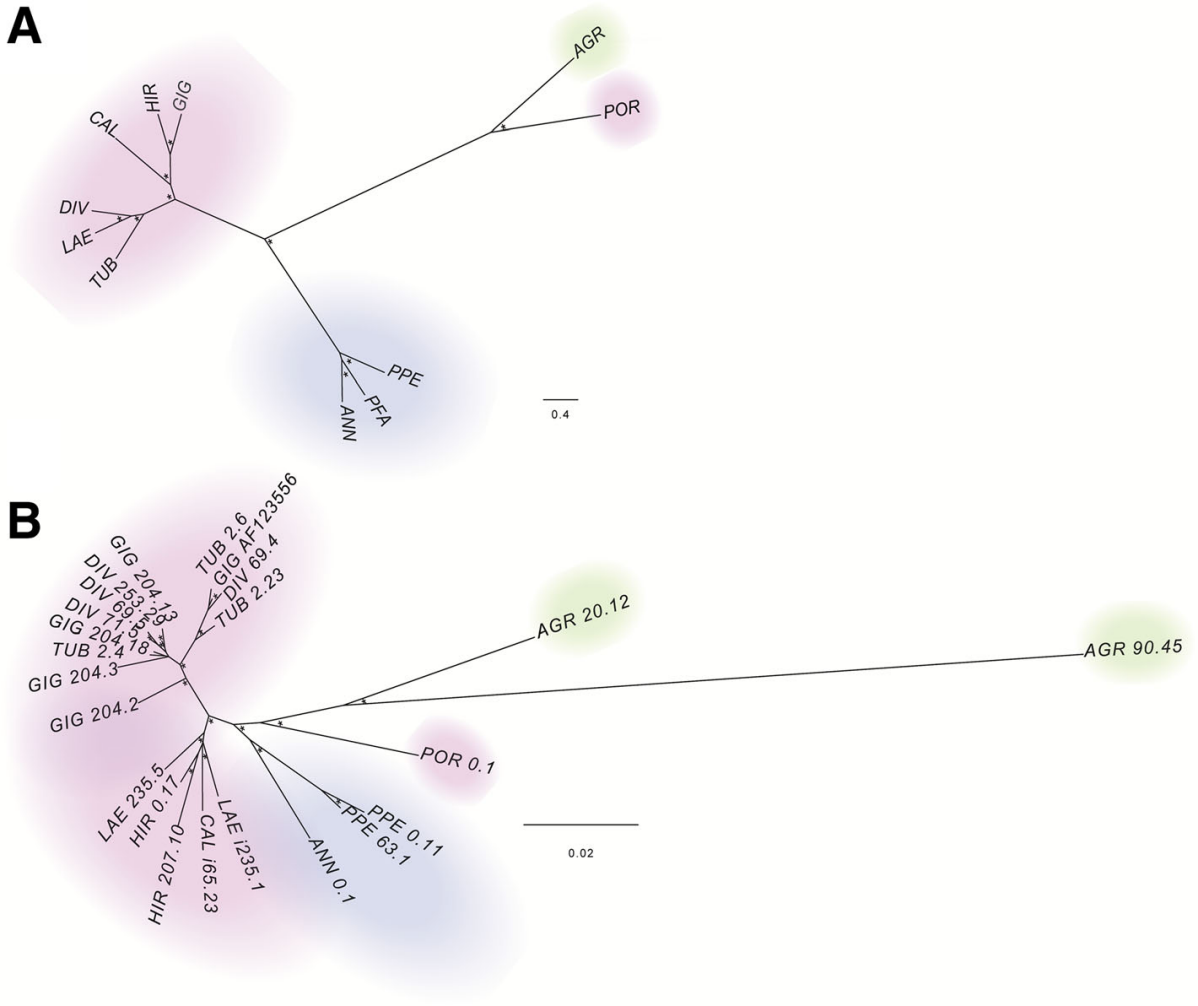
**a** Dendrogram obtained by a hierarchical clustering analysis based on genome proportion data of *Copia*- and *Gypsy*-related clusters (as obtained by hybrid clustering using RepeatExplorer) of different *Helianthus* species. Asterisks indicate multiscale bootstrap resampling (only values >60% are given). The bar represents the genetic distance. **b** Maximum composite likelihood phylogram of the same *Helianthus* species based on ETS sequences isolated by Timme et al. (2007). Asterisks indicate multiscale bootstrap resampling (only values >60% are given). The bar represents the genetic distance. Colours indicate the different analysed sections: pink for *Divaricati*, light blue for *Helianthus* and green for *Agrestis* section. Acronyms as defined in Table 1

The results of hierarchical clustering of all genome proportions data concerning LTR-RE *Copia* and *Gypsy* related clusters were compared with a phylogeny obtained with rDNA ETS sequences [24] (Fig. 2). The dendrogram (Fig. 2a) highlights a division within the genus *Helianthus*, supporting separation among the three different sections analysed, with perennial species of the *Divaricati* section and species of the *Helianthus* section occupying close branches of the tree and *H. agrestis* and *H. porteri* being more distant.

The two trees showed similar topologies, and the few differences observed concerned the *Divaricati* species. In fact, separation into distinct clades corresponding to the *Divaricati* subsections, as previously established [24], is not supported using LTR-RE genome proportion values (Fig. 2a). Furthermore, the occurrence of three distinct clades was more evident using LTR-RE genome proportion values than ETS sequences, suggesting that changes of the repetitive component have accentuated the differences among species.

Interestingly, both trees indicate a clear separation between annual and perennial species, with the annual *H. porteri* being separated from the other *Divaricati* species (which are perennial) and closer to the annual *H. agrestis*.

**Fig. 3:**
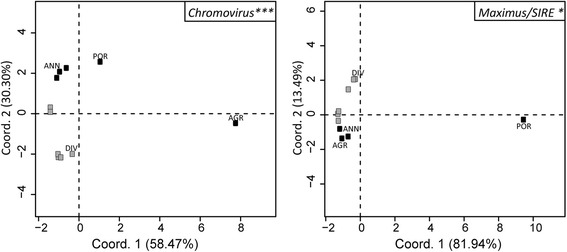
PCA plots of genome proportion values for *Gypsy* and *Copia* lineages with a percentage of reads >1% in annual (black dots) and perennial (grey dots) species. The percentage of variation accounted is reported on each axis. Asterisks mark permutational MANOVA significance with the following significance codes: 0.001 ‘***’ 0.05 ‘*’

Furthermore, principal component analysis of genome proportion of the most abundant *Copia* and *Gypsy* lineages, *Maximus/SIRE* and *Chromovirus*, showed a significant (*p* < 0.05) separation between annual and perennial species (Fig. 3).

### LTR-retrotransposon dynamics during *Helianthus* evolution

To gain insight into the evolution of LTR-REs within the genus *Helianthus*, amino acid sequences of the RT domain were isolated from individual clustering analysis and aligned to produce distance trees that allowed us to evaluate the relationship among LTR-RE lineages for *Gypsy* and *Copia* superfamilies. Both trees showed a clear-cut separation of RT-encoding sequences according to their lineage (see Additional file 2: Figure S2).

**Fig. 4:**
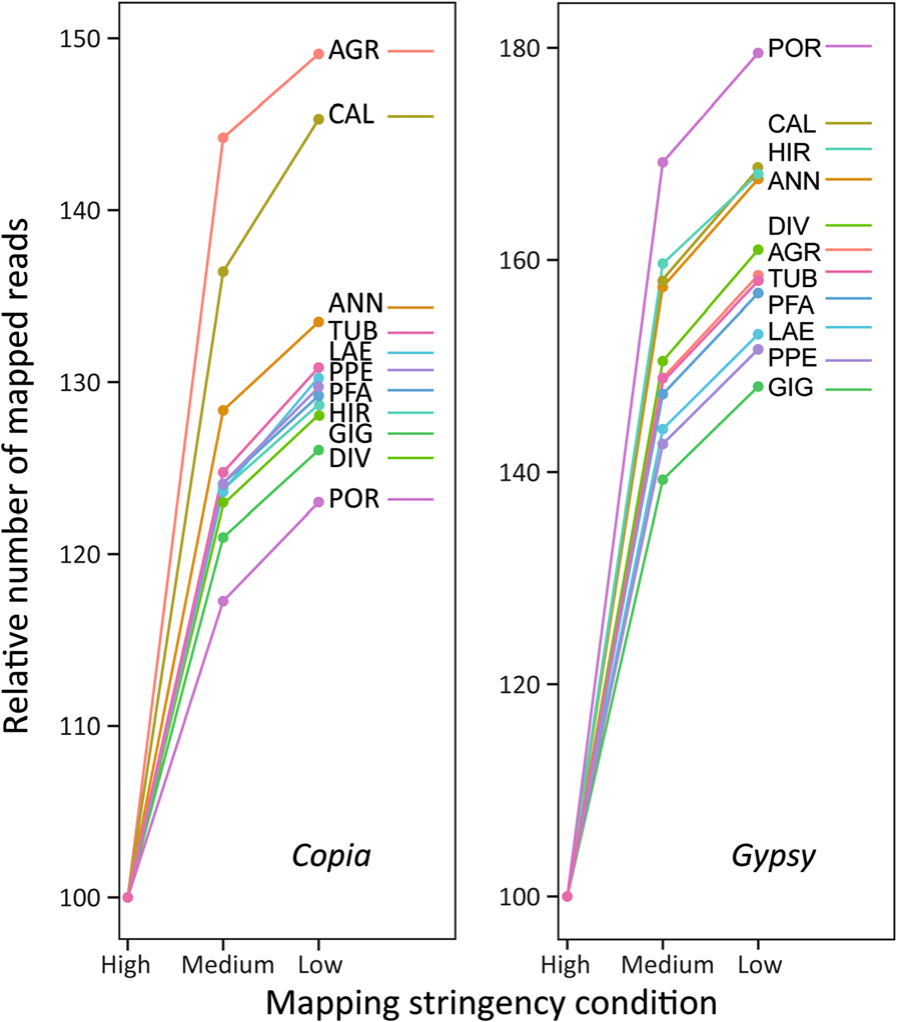
Number of mapped Illumina reads on sets of RT species-specific domains belonging to different lineages at different stringency parameters (see Methods). Acronyms as defined in Table 1

The comparative timing of LTR-RE proliferation was inferred analysing sequence conservation, by mapping Illumina reads to the DNA sequences encoding the RT domains of LTR-RE-related clusters at different stringency conditions (Fig. 4). The more a sequence is conserved, the more recent should be the proliferation of the related element. Overall, results showed that *Copia* RT domains of *H. agrestis* and *H. californicus* are the most divergent and those of *H. porteri* are the most conserved ones. In contrast, *Gypsy* elements showed similar sequence conservation among species, with the exception of *H. porteri*, whose RT domains were highly divergent.

The previously reported comparative analysis (Table 5) also allowed us to infer both the evolutionary trend of each LTR-RE lineage and relationships among the species of the genus *Helianthus*. Separated clusters belonging to the same lineage presumably represent different sublineages according to their sequence similarity. Through hierarchical clustering analysis of LTR-RE clusters, based on the genomic proportion of each cluster, we identified and quantified groups of homologous clusters sharing similar abundance levels between the species [37].

**Fig. 5:**
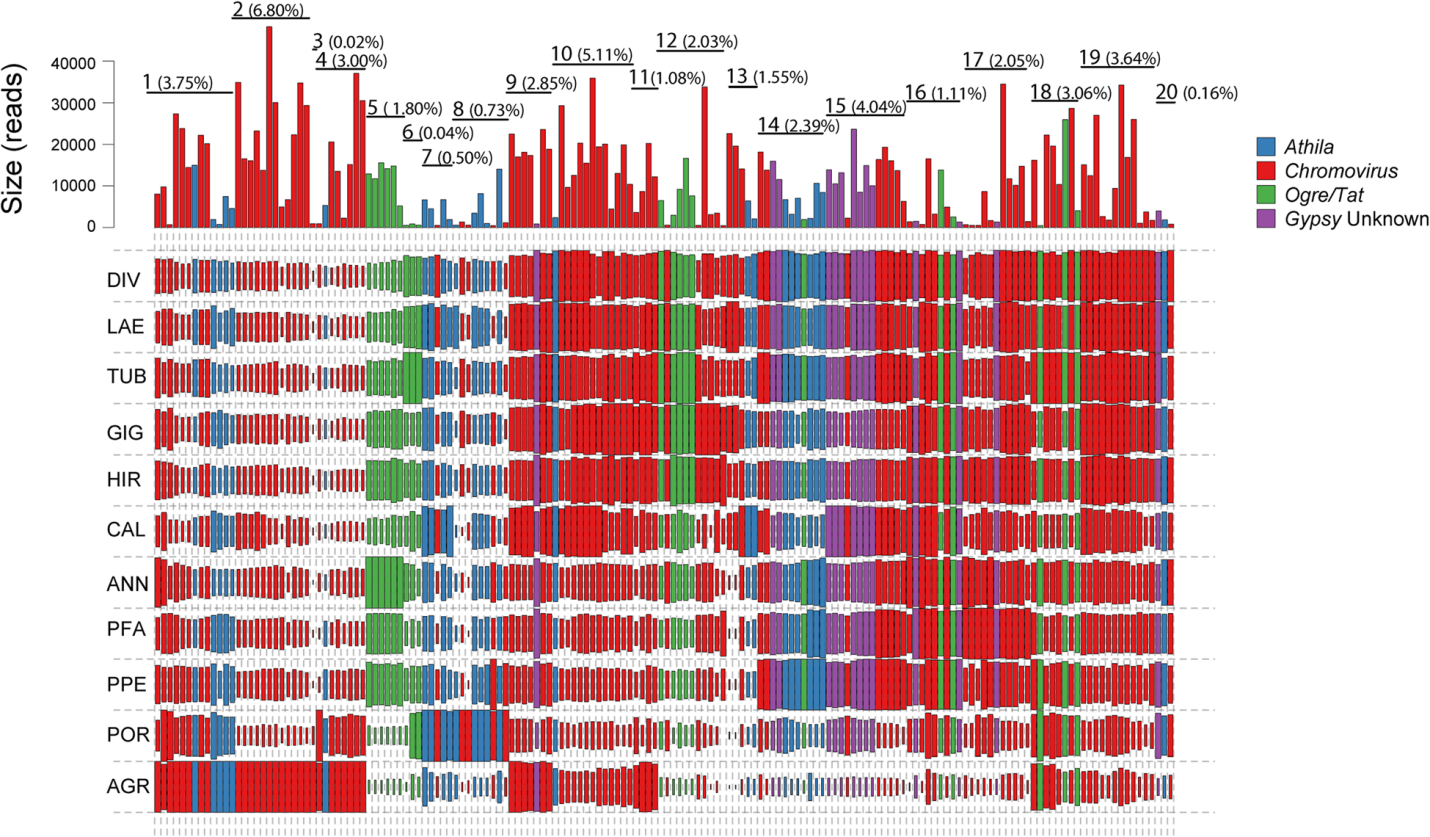
Sequence composition of the LTR-*Gypsy*-RE-related clusters. The size of the rectangle is proportional to the genome proportion of a cluster for each species (acronyms as in Table 1). Bar plot in the top row shows the size of the clusters as number of reads in the comparative analysis. Upper lines label groups of clusters as assessed by a hierarchical clustering of the results. The percentage of reads included in the group is shown in parentheses. The colour of the rectangles corresponds to the lineage of the *Gypsy* LTR-RE

The genome proportion of homologous clusters belonging to *Gypsy* superfamily, in the 10 species and one subspecies analysed, is reported in Fig. 5 (for the *Copia* superfamily see Additional file 3: Figure S3). Clusters were in turn grouped according to their abundance among the 11 genotypes: each group represents clusters showing a similar pattern of abundance.

*Gypsy*-related clusters can be subdivided into 20 groups, representing from 0.02 to 6.80% of the genome (Fig. 5). All but one sublineage of *Gypsy* LTR-REs were represented in all species; this sublineage, which belongs to group 12, was abundant in the genome of all species and absent in *H. porteri* and in *H. agrestis*. It is evident from Fig. 5 that *Gypsy* sublineages showed different patterns among the 10 species and one subspecies, indicating that each group has experienced a different evolutionary dynamics. For instance, group 2 clusters (belonging to *Chromovirus* lineage), the most abundant in terms of genome proportions counting 6.80% of analysed reads, were especially represented in *H. agrestis.* Similarly, group 3, which had only one C*hromovirus*-related cluster, was highly specific for this species, being barely represented in the others. On the other hand, group 10, a group made of *Chromovirus* sublineages, which was the second most prominent group with regard to genome proportion, was overrepresented in perennial species compared to annuals.

In the absence of whole genome sequences of each species, we compared the tendency to produce solo-LTRs among the selected species by measuring the ratio between the average coverage of the LTR and the respective RE-coding portion of 41 full-length LTR-REs isolated from available genome scaffolds of *H. annuus* (Additional file 4: Table S1).

If all elements of a RE sublineage were intact, the average coverage of the 5′-LTR should be two-fold that of the respective inter-LTR DNA sequence. Ratios >2 should indicate the occurrence of solo-LTRs of that RE sublineage. On average, these ratios ranged from 0.0003 to 4.10, with only 3 out of 41 REs showing a ratio > 2, i.e. unequal homologous recombination should not be very common in sunflowers.

**Fig. 6:**
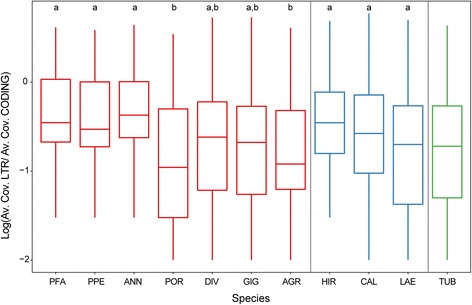
Distribution (on a logarithmic scale) of the ratio between the average coverage of 5′-LTR and respective coding (inter-LTR) DNA sequence related to the 41 isolated REs grouped per species. Species are distributed by increasing genome size keeping separated ploidy levels. Diploid species are in red, tetraploid in blue and hexaploid in green. The boxes represent the 25–75%, whiskers the whole range of values and dots the outliers. The lines in the boxes represent the medians of the distributions. Within diploid or tetraploid species, those indicated by different letters are significantly different (*p* < 0.05) according to Tukey’s test

In Fig. 6, the distribution of ratios between average coverage of LTR and inter-LTR region of 41 REs in the species and subspecies is reported, keeping separated diploid, tetraploid and hexaploid species. Considering diploid species, significant differences occurred, indicating that some species should have accumulated more solo-LTRs than other. Interestingly, the highest values were measured in the species with the smallest genomes, suggesting that unequal homologous recombination occurred, leading to a reduction of genome size (Fig. 6). The negative correlation between LTR/inter-LTR average coverage ratio and genome size was significant for *Gypsy* elements (see Additional file 5: Figure S4).

### Genome doubling without polyploidization: *H. agrestis*

Relative 4C DNA absorption analysis (Table 2) indicated that *H. agrestis* genome size was almost two-fold larger than expected in a diploid species. The expansion of its genome is supported, at least in part, by the huge genome proportion of *Gypsy-Chromovirus*-related clusters (Table 5) compared to the other analysed species.

**Fig. 7:**
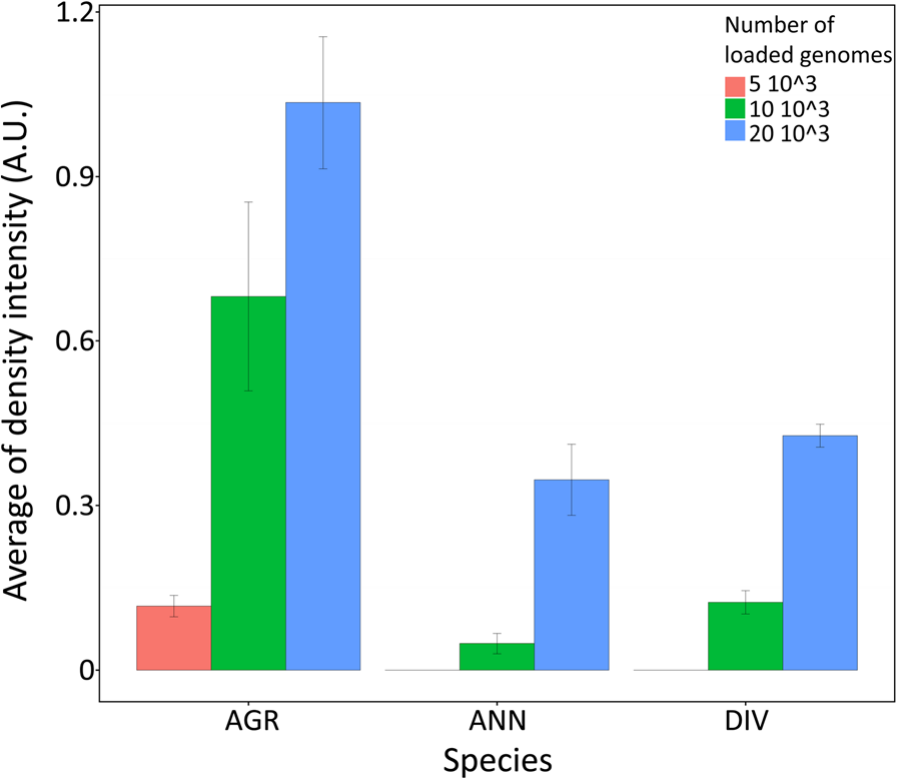
Histograms obtained by the densitometric scanning of slot blots of genomic DNAs of *H. agrestis* (AGR), *H. annuus* (ANN) and *H. divaricatus* (DIV), hybridized with a *H. annuus* probe, consisting in a DNA fragment belonging to a *Gypsy*-*Chromovirus* element. Each value is the mean (± SE) of three replicates

The involvement of *Chromoviruses* in the genome expansion of *H. agrestis* was confirmed by dot-blot hybridization experiments using a *Chromovirus* sequence isolated from *H. annuus* DNA as probe. The results of hybridization are reported in Fig. 7. It can be observed that the hybridization intensity in *H. agrestis* is more than two-fold that of *H. annuus* and *H. divaricatus*. The copy number of the analysed sequence in *H. annuus* amounted to 1600 per haploid genome, while in *H. agrestis*, it amounted to 4700. Considering that the probe used in this experiment was heterologous to *H. agrestis*, it can be hypothesized that the copy number in this species is even underestimated.

A neighbour-joining phylogenetic tree based on multiple alignment of sequences of *Chromovirus*-RT amino acid sequences of *H. agrestis* and RT amino acid sequences of *Chromoviruses* of several species (i.e. *Beta vulgaris, Zea mays, Vitis vinifera*) was performed to define the specific *Chromovirus* clade(s) to which the largely abundant *Chromoviruses* of *H. agrestis* belonged. The tree indicated that most sequences of *H. agrestis* belonged to *Tekay*-related elements, while *Galadriel* and other clades were barely represented (data not shown).

**Fig. 8:**
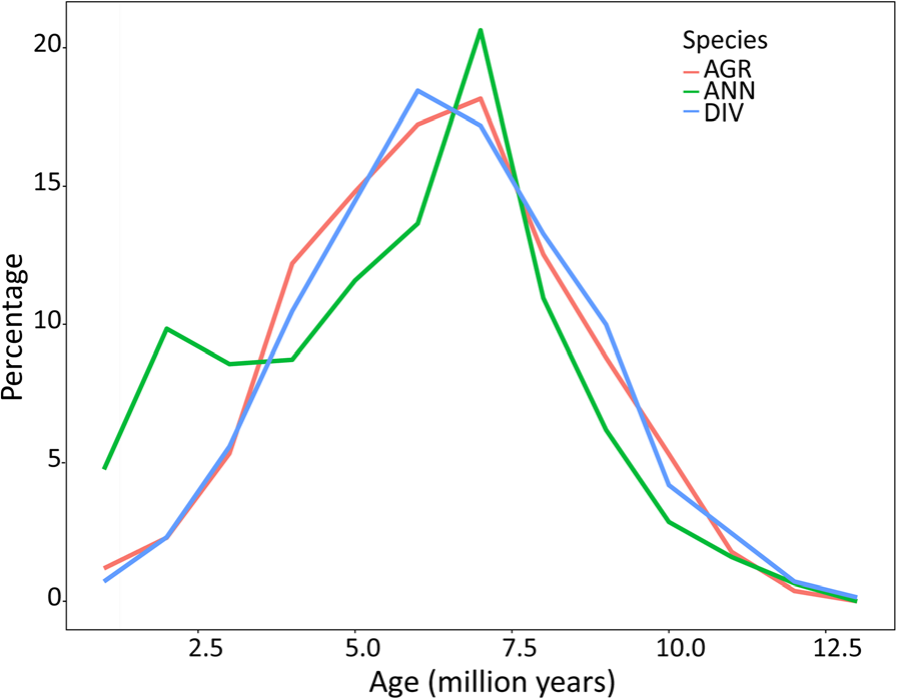
Timing of the *Chromovirus* retrotranspositional activity in *H. agrestis* (AGR), *H. annuus* (ANN) and *H. divaricatus* (DIV). The y axis shows the percentage of the total pairwise comparisons of reads matching the *Chromovirus* RT domain in the three species

Finally, Illumina reads matching a *Chromovirus* RT-encoding domain of *H. agrestis*, *H. annuus* and *H. divaricatus* were pairwise compared and their distances [38] were converted into insertion dates according to SanMiguel et al. [39] and Piegu et al. [8], but using a mutation rate of 2 × 1 0^-8, i.e. 2-fold that calculated for sunflower gene sequences, to keep into account that mutation rate of retrotransposons is higher than that of genes, as it depends on error-prone action of reverse transcriptase during element retrotransposition besides on mutations occurring across generations. This analysis enabled the identification of one retrotranspositional wave, mostly overlapping in terms of time in the three species (Fig. 8). Although timing data should be taken cautiously, the proliferation burst should have started 10 MYA and reached its apex around 6-6.5 MYA. *Chromovirus* amplification has nearly ceased in *H. agrestis* and *H. divaricatus*, while an additional recent and smaller amplification peak was observed in *H. annuus* (Fig. 8). Because of the much larger proportion of *Chromoviruses* observed in *H. agrestis* than in the other two species (Table 5), it can be deduced that amplification of these elements occurred concurrently in the three analysed species, with large differences in the amplification rate, which was much higher in *H. agrestis* than in *H. annuus* and *H. divaricatus*.

## Discussion

### Interspecific variation of LTR-RE abundance

The first goal of our research was to establish the extent of interspecific repetitive DNA variation related to LTR-RE abundance. Analysis of the genome size showed considerable variations among species, even in those with the same chromosome number. Conversely, comparative clustering of Illumina reads among species provided information about an “average” composition of this genus. Repetitive DNA represented 77.5% of this “metagenome”, similar to that already reported for *Helianthus annuus* [40]. Among species, the repetitive DNA ranged from 73.6% of the genome in *H. petiolaris* ssp. *fallax* to 84.2% in *H. agrestis*, i.e. it showed relative uniformity. Genome structure was also similar among the analysed species, with LTR-REs representing the vast majority of repetitive sequences.

Genome size variations in species with the same chromosome number are usually attributed to variations in the abundance of repetitive DNA [41]. Concerning LTR-REs, differences in abundance may be derived from massive amplification through retrotransposition or from DNA loss by unequal homologous recombination, which produced solo-LTRs [2].

As a result of the amplification burst(s) that may have occurred, our data on RT sequence conservation evidence that, for some elements, RE amplification have occurred in different times for different species. For instance, in *H. porteri,* the great abundance and the low sequence variability of *Copia* elements should imply that amplification of these REs has occurred in a more recent past than for the other species. On the contrary, the low number and large sequence variability of *Gypsy* elements indicates that, in *H. porteri*, proliferation of such elements ceased earlier than in the other species.

On the other hand, although indicating that solo LTRs (generally produced by unequal homologous recombination) are not common in sunflowers, differences in the extent of DNA removal can be inferred by our data. In fact, the ratio between the average coverage of the LTR and the respective RE-coding portion of 41 full-length LTR-REs suggest that diploid species of the *Helianthus* section show a higher frequency of putative solo-LTRs and hence, presumably, a stronger tendency to unequal homologous recombination compared to diploid *Divaricati* species, which contributed to the reduced genome size of the former species than of the latter.

We observed striking differences analysing the relative abundance of LTR-REs, from the superfamily to the sublineage level. These results suggest that the common ancestor of *Helianthus* contained different LTR-RE sublineages and that, after species separation, such sublineages were subjected to different rates of amplification/loss, while no new LTR-RE sublineage originated (by mutations or by horizontal transfer) in the genome.

One common feature of *Helianthus* genomes was the abundance of *Gypsy* elements, which was always higher than that of *Copia* ones, as observed in the cultivated sunflower (see [40]). However, it is to be noted that the ratio between *Gypsy* and *Copia* abundance is highly variable. Such variability is very large when compared to the observed interspecific variation of this ratio in all angiosperm species, ranging from 5:1 in the genome of papaya to 1:2 in that of grapevine (see [11, 42]).

Our data evidence the impact of RE dynamics on speciation within the genus *Helianthus*. In fact, it can be speculated that the huge variations observed have negatively affected chromosomal colinearity, favouring the reproductive isolation of the species.

### LTR-RE abundance and evolution in *Helianthus*

The second point of our study was to investigate the relationship between changes of LTR-RE abundance and the evolution of the genus.

The different abundance of LTR-RE lineages was used to estimate the evolutionary relationship among the different species. Compared to the last reported *Helianthus* phylogeny [24], the LTR-RE genome-proportion-based dendrogram parallels *Helianthus* evolution in keeping the *Helianthus* and *Agrestis* sections separated. Although REs could be considered as autonomous entities, these data suggest that LTR-REs and their hosts in the genus *Helianthus* have generally evolved together.

However, results also indicate a firm separation between *H. porteri* and the other species of the *Divaricati* section, while clades within the branch corresponding to perennial species of the *Divaricati* section are poorly supported, confirming [28] that LTR-REs have continued to evolve during speciation. If one compares the two trees, although their topologies are very similar, genetic distances are larger using LTR-RE genome proportion values than rDNA-ETS sequences, suggesting the involvement of LTR-RE amplification/loss in increasing genetic differentiation between species.

### LTR-RE abundance and annual or perennial habits of a species

The third point was to investigate whether there is a connection between annual or perennial habits of species and LTR-RE abundance. An interesting aspect of our analyses is that, considering only diploid species, the genome size of annual sunflowers is smaller than that of perennials, with the exception of *H. agrestis* (belonging to the monophyletic section *Agrestis*). This result is in line with cytophotometric determinations of genome size of *Helianthus* species previously provided [33]. Also, Southern blot hybridization analyses of *Gypsy* and *Copia* elements [25, 26, 43] evidenced a clear-cut separation between annual and perennial species.

The present data reinforce the hypothesis that LTR-RE abundance is more affected by species habit (i.e. annual vs. perennial) than by taxonomic relationships among species in determining the accumulation (or the reduction) of specific LTR-RE superfamilies or lineages. Keeping separated *Gypsy* and *Copia* lineages, PCA showed a significant separation between annuals and perennials for the most abundant lineages of both the *Gypsy* (*Chromovirus*) and the *Copia* superfamily (*Maximus/SIRE*). *Chromovirus* LTR-REs were more abundant in perennial species; *Maximus/SIRE* elements were more abundant in annuals. Such separation was not significant analysing the other LTR-RE lineages, suggesting that habit affected (with different outcomes) the abundance of specific types of LTR-REs.

The reasons for such differences between annual and perennial species can be only speculated. They might have established casually during *Helianthus* evolution. Another possibility is that the influence of the habits on RE abundance might be related to the different life cycle length between perennial and annual species. In fact, in case of RE proliferation or unequal homologous recombination burst, at each generation, the number of LTR-RE can be fixed and in the subsequent generation, new insertion/deletion events can occur, adding or losing other elements. Moreover, changes in LTR-RE abundance can be related to recombination events by which homologous chromosomes with high or low numbers of elements co-segregate in the subsequent generations.

The additive accumulation or loss of LTR-REs at each generation and the effect of co-segregation of chromosomes with increased or decreased numbers of LTR-REs change the number of these elements in the genome. The occurrence of both processes may be related to the number of generations in which accumulation or loss occur, that is larger in annuals than in perennials. Further studies, in other plant genera, are necessary to establish the occurrence of a relationship between habit and transposon accumulation and the reasons for which such a relationship occurs.

### Coevolution of LTR-RE lineages and sublineages between and within species

The fourth aim was to determine the degree of coevolution of different LTR-RE lineages or sublineages between and within species.

At lineage level, *Gypsy Chromovirus* elements are by far the most abundant in all species, suggesting that probably the amplification of these LTR-REs largely predated species divergence in this genus. These variations indicate that high amplification rate was maintained in some species (as *H. agrestis*) even after speciation or that chromosome rearrangements, such as large duplications or deletions (often driven by retrotransposons, [2]) might have occurred. However, the large conservation of RT-domain sequences among species indicate that if LTR-RE lineages may have experienced amplification or loss during or after *Helianthus* speciation*,* these events have occurred in relatively recent times.

At sublineage level, the occurrence of different groups of *Gypsy* and *Copia* REs, with different patterns of abundance among species, reinforces the view of a very large variability in the abundance of LTR-RE sublineages originated after species separation.

Our data also point out the necessity of referring to single sublineages when studying retrotransposons, in fact, evaluating differences at superfamily or even at lineage level does not account for the existence of large differences among sublineages within a single lineage. Coevolved groups of LTR-RE in sunflowers are made of elements belonging to different lineages and, on the other hand, sublineages of the same lineage belong to groups with different evolutionary trends.

### The impact of LTR-RE proliferation on genome size: the case of *H. agrestis*

We studied variations in the dynamics (amplification, loss, proliferation dating) of a specific LTR-RE lineage, *Chromovirus*, among species. We observed that *H. agrestis*, a diploid species, has a genome size more than two-fold that of the largest genome sized diploid species. This huge genome expansion is, at least in part, related to amplification of LTR-REs of the *Chromovirus*-*Tekay* lineage, as indicated by the average coverage of *Chromovirus*-related clusters and by slot-blot hybridization experiments. Such huge expansion is similar to others reported in different plant species, as *Vicia pannonica* [7] and *Oryza australiensis* [8]. As discussed before, it is possible that in *H. agrestis*, beside proliferation of *Chromovirus* LTR-REs, duplications of chromosomal regions and co-segregation of homologous chromosomes with high number of LTR-REs have also contributed to hugely increase the genome size.

Analysis of conservation of *Gypsy* RT-encoding sequences showed similar levels of conservation in all species, excluding *H. porteri*. This suggests that *Gypsy* proliferation bursts have occurred in the same period in all analysed species except *H. porteri*. Dating proliferation burst of *Chromovirus* LTR-REs also showed that it occurred in the same period in the three analysed species (*H. agrestis*, *H. divaricatus* and *H. annuus*). It can be inferred that, in that time span, proliferation of *Chromoviruses* in *H. agrestis* was by far more active than in the other species. Interestingly, proliferation seems to be exhausted in *H. agrestis* and *H. divaricatus* while it is continuing in *H. annuus*, again indicating the peculiarity of LTR-RE dynamics among species.

## Conclusions

Our study exploits the potentiality of massively parallel sequencing technologies applied to the analysis of genome structure and evolution. It shows a great variability of LTR-RE abundance at superfamily, lineage and sublineage levels and suggests that the evolution of the LTR-RE component of the genome in *Helianthus* species is partly independent of the evolution of such species. This is not surprising, because LTR-REs are (at least potentially) autonomous in their reproduction [9]. Indeed, cases of species-specific huge amplification of LTR-RE lineages were already known, even in sunflowers [30, 44], and LTR-REs were still active in retrotransposition in *H. annuus* [45].

The availability of the forthcoming reference genome for *H. annuus* [46] in conjunction with new sequencing technologies, allowing for the production of very long DNA sequences, will be useful for further elucidating many aspects of genome evolution in this genus.

## Methods

### Data availability

Whole-genome shotgun sequences described are available on NCBI Sequence Read Archive under the accession numbers SRR5804988 (*H. agrestis*), SRR2919251 (*H. annuus*), SRR5713981 (*H. petiolaris* ssp. *fallax*), SRR5713980 (*H. petiolaris* ssp. *petiolaris*), SRR5804989 (*H. porteri*), SRR5713976 (*H. divaricatus*), SRR5713977 (*H. giganteus*), SRR5713975 (*H. californicus*), SRR5713978 (*H. hirsutus*), SRR5713979 (*H. laevigatus*), SRR5713974 (*H. tuberosus*). Clusters and contigs assembled by RepeatExplorer are available at the Sequence Repository Page of the Department of Agriculture, Food, and Environment of the University of Pisa (http://pgagl.agr.unipi.it/sequence-repository/).

The *Chromovirus*-related sequence used as probe in molecular analyses is available on the NCBI website (https://www.ncbi.nlm.nih.gov/) under the accession number MF448448.

### Plant materials and DNA isolation

The *Helianthus* species used are listed in Table 1. Seeds were obtained from USDA, ARS, National Genetic Resources Program, USA (ARS-GRIN) (https://npgsweb.ars-grin.gov/gringlobal/search.aspx). Seeds were germinated in moistened paper in Petri dishes and 2-3 cm long plantlets were grown in pots in the greenhouse. Leaf tissue was sampled from single individuals of each genotype and total genomic DNA was extracted using the CTAB procedure [47].

### Cytological analyses

Primary and secondary root apices were collected from three plantlets per species, treated with 2 mM 8-hydroxyquinoline for 90 min and fixed in ethanol: acetic acid (3:1, *v*/v). The roots were washed in an aqueous solution of 6 mM sodium citrate plus 4 mM citric acid, treated with a mixture of 3% pectinase (Sigma), 4.5% cellulase (Calbiochem) and 0.5% cellulase Onozuka (Serva) in citrate buffer pH 4.6 for 15 min at 37 °C and subsequently squashed under a coverslip in a drop of 60% acetic acid. The coverslips were removed after freezing at −80 °C. The preparations were air-dried and Feulgen-stained after hydrolysis in 1 N HCl at 60 °C for 8 min. After staining, the slides were subjected to three 10-min washes in SO_2_ water prior to dehydration and mounting in distyrene-dibutylphthalatexylene (DPX; BDH Chemicals). Feulgen DNA absorptions in individual prophase nuclei were measured in images captured by a charge-coupled-device camera on a Leica DMRB microscope, using a Leica Q500MC image analyser. On the same slides, metaphase plates were scored to determine chromosome number.

### Illumina sequencing

The DNA samples were sheared into fragments for sequencing. Paired-end (insert size) libraries were prepared as recommended by Illumina (Illumina Inc., San Diego, CA), with minor modifications. Paired reads were first tested for quality and trimmed at 90 nt in length, using Trimmomatic [48] with the following parameters: ILLUMINACLIP:2:30:10 CROP:90 MINLEN:90, to remove adapters and low-quality regions. All reads containing organellar DNA sequences were removed using CLC-BIO Genomic Workbench 7.0.4 (CLC-BIO, Aarhus, Denmark), against chloroplast and mitochondrial sequences of *H. annuus* (NCBI reference sequence NC_007977 and KF815390, respectively).

### Identification of LTR-RE sequences

In order to perform a comparative analysis of the repetitive component of 10 species and one subspecies of the genus *Helianthu*s, a hybrid graph-based clustering method (RepeatExplorer, [36]) was applied allowing de novo identification of repeats and their proportion in each genome. Accordingly, a random set of sequences composed of reads of each species was used, choosing a number of reads proportional to the ploidy level of the species to ensure that the clusters obtained were comparable.

RepeatExplorer output was filtered to collect the clusters identified as REs. To increase the number of such clusters, similarity searches on the remaining unknown clusters were performed by BLASTN and tBLASTX against a library of sunflower repetitive sequences, SUNREP [27]. All annotated clusters were collected to prepare an in-house reference library of *Helianthus* LTR-REs.

Additionally, de novo identification of full-length LTR-REs was performed on 40 genome scaffolds of *Helianthus annuus*, downloaded from the NCBI website (www.ncbi.nlm.nih.gov/; Additional file 4: Table S2), by searching structural features with LTR-FINDER [49] and DOTTER [50]. All putative LTR-REs were annotated using BLASTX and BLASTN against the nr database of NCBI and transferring the annotation from the best hit.

### Mapping procedure for abundance estimation

Abundance values of sequences were estimated for each species by counting the total number of reads (per million) mapping to cluster sequences. This method has already been used for many plant species [42, 51–53], including sunflower [27, 31]. CLC-BIO Genomic Workbench was used to perform mapping at high stringency with the following parameters: mismatch cost = 1, deletion cost = 1, insertion cost = 1, similarity = 0.9 and length fraction = 0.9.

In another analysis, to estimate the occurrence of solo-LTRs in all the species, every read set was mapped onto each isolated full-length RE, keeping separated the 5′-LTR region and the inter-LTR one.

### Phylogenetic trees

All species were analysed one-by-one using RepeatExplorer to perform graph-based clustering on a random set of genomic sequences. Subsequently, the protein domain tool was used to identify and extract conserved regions of RT protein domains for *Gypsy* and *Copia* RE superfamilies.

Afterwards, the multiple protein alignment was calculated using Clustal Omega [54] and the phylogenetic trees were built using a neighbour joining clustering method (NJ) (1000 bootstrap replications).

In another analysis, the external transcribed spacer of ribosomal DNA (rDNA-ETS) sequences reported in Timme et al. [24] were used to draw a dendrogram of the species used in this work. An aligned data set was prepared for phylogenetic analyses concatenating the 5′ and 3′ single copy regions of the each ETS into one partition after removing subrepeats (as described in [24]). The alignment was performed using CLUSTAL X [55] and the phylogenetic trees were built using NJ (1000 bootstrap replications).

Finally, a dendrogram based on the genome proportions data of each LTR-RE analysed was built by using R package pvclust version 1.3-2 [56], which allowed the assignment of the uncertainty in hierarchical cluster analysis (10,000 bootstrap replications).

### Retrotransposon insertion time analysis

For a comparative estimation of the age of each RE lineage the sequence conservation of the RT domain was analysed in all species. Illumina reads were mapped onto RT domain encoding sequences and counted using CLC-BIO Genomic Workbench at different stringencies (high, medium or low). We kept fixed mismatch cost, deletion cost and insertion cost at 1, changing similarity and length fraction at 0.9, 0.7 or 0.5, respectively. The ratio between the number of reads mapping onto a given lineage at medium and high stringencies reflects the sequence conservation level of the elements that belong to that lineage: assuming similar evolutionary rates in each lineage, the lower the ratio, the higher the sequence conservation.

Timing of *Chromovirus* LTR-REs proliferation bursts in *H. agrestis*, *H. annuus* and *H. giganteus* were also estimated according to Piegu et al. [8] and Ammiraju et al. [57] through analysis of the distribution of divergence values between pairwise comparisons of sequences belonging to the same lineage.

After collecting *Chromovirus* RT domain-related sequences (90 nt-long) from RepeatExplorer, cluster mapping was performed using CLC-BIO Genomic Workbench for isolating reads homologous to RT in each species or subspecies. Then, paralogous reads were pairwise compared (using MEGA version 7; [58]) within each species or subspecies and Kimura distances [38] were calculated. Kimura distances were converted to millions of years ago (MYA) using a substitution rate of 2*10-8, i.e. two-fold the value determined for gene sequences of sunflower as already used for sunflowers [30, 59]. In fact it is to be noted that, beside accumulating mutations as time passes, REs accumulate further mutation during retrotranscription, being the RT error-prone [10]. Hence, an increased substitution rate is to be used for calculating retrotransposition time periods.

### Dot blot hybridization and calculation of sequence copy number

A 678 bp-long *Gypsy* fragment was amplified by PCR from 50 ng *H. annuus* genomic DNA. Primers were designed onto an integrase encoding sequence (forward primer: 5′-AAACGGATGGACAAACTGAACG-3′) and a chromodomain (reverse primer: 5′-CCTTGACTATGCGAATCTTGCT-3′) of a *Chromovirus*-related cluster from the hybrid graph-based clustering. The PCR conditions were as follows: at 94 °C for 4 min, then 30 cycles of 94 °C for 30 s, 58 °C for 30 s, 72 °C for 40 s. Final extension was performed at 72 °C for 7 min. The PCR products were purified with Wizard SV gel and PCR clean-up system (Promega) and cloned into the pGEM-T Easy plasmid vector (Promega). The cloned fragments were sequenced and one clone was selected (EMBL accession number MF448448).

Dot blot was prepared by applying dilution series (three replicates) of DNA to positively charged nylon membranes (Roche) using a Bio-Dot apparatus (Biorad). Based on 4C Feulgen absorptions and using a *H. annuus* C-value estimation of 3.3 pg [20], *H. agrestis*, *H. annuus* and *H. divaricatus* denatured genomic DNAs were spotted in a dilution series from 20 × 10^3 to 5 × 10^3 genomes. Similarly, dilutions of denatured PCR product of the *Chromovirus* fragment of 678 bp, were applied to filters in a dilution series representing 5 × 10^7 to 0.625 × 10^7copies.

The probe used for hybridization was digoxigenin-labelled by PCR using 1× PCR buffer, 0.5 μl *Taq* DNA polymerase (Promega), dNTP labelling mix (final concentrations 200 μM dATP, 200 μM dCTP, 200 μM dGTP, 190 μM dTTP, 10 μM digoxigenin-11-dUTP, alkaline labile; Roche), 2.5 mM MgCl2, 0.8 μM each forward and reverse primers, 1 ng plasmid DNA derived from selected clone as template (total volume 50 μl). Samples were heated at 94 °C for 4 min and the PCR reaction was performed as described above. The digoxigenin-labelled PCR product was purified with Wizard SV gel and PCR clean-up system (Promega).

Hybridization was performed using 15 ng/ml probe at 65 °C for 12 h in deionized water, 5 × SSC, 2% blocking reagent (Roche), 0.02% SDS and 0.2% SLS. The filter was washed twice in 2 × SSC, 0.1% SDS for 15 min at room temperature, once in 1 × SSC 0.1% SDS for 30 min at 68 °C and once in 0.5 × SSC, 0.1% SDS for 30 min at 68 °C. The temperature of the final wash was calculated in order to ensure hybridization of DNA sequences sharing at least 85% similarity with the probe. Detection was performed using the DIG-Nucleic Acid Detection Kit (Roche) according to the manufacturer’s instructions.

Finally, the membrane was scanned densitometrically using a UVP System 5000 equipped with GelBase-GelBlot software. Estimation of the copy number of the sequence probed in the genomic DNA was carried out as described previously [25].

### Statistical analyses

Genomic proportions of the most abundant *Gypsy* and *Copia* RE lineages were subjected to principal component analysis (PCA) using the implementation of the R package FactoMineR version 1.26 [60] and to permutational MANOVA [61] with R package vegan version 2.0-10 [62]. Differences among average coverage of LTR over coding region ratios were tested by using the non-parametrical method of Tukey. A separate test was performed for each group of species with the same ploidy level.

## Additional files

Additional file 1: Figure S1. Correlation plot between genome proportion and total read count per million reads related to the 248 clusters annotated as LTR-REs (acronyms as in Table 1). (PDF 222 kb)

Additional file 2: Figure S2. Distance tree of LTR-Copia RT domains of 10 species and one subspecies of Helianthus subjected to NJ analysis. Bootstrap values higher than 0.6 are shown with asterisk. Bar represents the nucleotide distance. Outgroups are RT domains of other species. Distance tree of LTR-Gypsy RT domains of 10 species and one subspecies of Helianthus subjected to NJ analysis. Bootstrap values higher than 0.6 are shown with asterisk. Bar represents the nucleotide distance. Outgroups are RT domains of other species. (PDF 487 kb)

Additional file 3: Figure S3. Sequence composition of LTR-Copia-RE-related clusters. The size of the rectangle is proportional to the genome proportion of a cluster for each species (acronyms as in Table 1). Bar plot in the top row shows the size of the clusters as number of reads in the comparative analysis. Upper lines label groups of clusters as asses by a hierarchical clustering of the results. The percentage of reads included in the group is shown in parentheses. The colour of the rectangles corresponds to the lineage of the Copia LTR-RE. All Copia-related repeats were shared among all the 10 species and one subspecies, with some peculiarities. In fact, differences were found even within lineages, producing 14 groups of RE sublineages with different abundance patterns, which accounted for 0.03 to 3.53% of the genome. For example, group 10, although being the most abundant (3.53% of the genome on the whole), was made up by nine clusters (eight annotated as Maximus/SIRE and one as Copia-unknown), that were represented in all species, but showed the largest abundance in *H. porteri*. On the contrary, the eight sublineages of group 12, annotated as Maximus as well, showed the highest genome proportions especially in *H. agrestis* and, to a lesser extent, in the Helianthus section. (PDF 454 kb)

Additional file 4: Table S1. List of 40 genomic scaffolds of Heliantus annuus downloaded from NCBI. **Table S2.** LTR RE-coding portion of 41 full-length LTR-REs isolated from available genome scaffolds of *H. annuus.* (XLS 373 kb)

Additional file 5: Figure S4. Distribution (on a logarithmic scale) of the ratio between the average coverage of 5′-LTR and respective coding (inter-LTR) DNA sequence related to 25 Copia (left) and 16 Gypsy (right) isolated REs, grouped per species. Species are distributed by increasing genome size keeping separated ploidy levels. Diploid species are in red, tetraploid in blue and hexaploid in green. The boxes represent the 25–75%, whiskers the whole range of values and dots the outliers. The lines in the boxes represent the medians of the distributions. Within diploid or tetraploid species, those indicated by different letters are significantly different (*p* < 0.05) according to Tukey’s test. (PDF 426 kb)

## Abbreviations

CTAB: Cetyl trimethyl-ammonium bromide
ETS: External transcribed spacer
LTR: Long-terminal-repeat
MANOVA: Molecular analysis of variance
MYA: Millions of years ago
NJ: Neighbor joining
ORF: Open reading frame
PCA: Principal component analysis
PCR: Polymerase chain reaction
RE: Retrotransposon
RT: Reverse transcriptase
SDS: Sodium dodecyl sulfate
SSC: Saline-sodium citrate
TE: Transposable element
USDA: United States Department of Agriculture
