# Supplementary material for: Genome-wide analysis of LTR-retrotransposon diversity and its impact on the evolution of the genus Helianthus (L.)
Source: BMC Genomics. 2017 Aug 18;18:634. doi: 10.1186/s12864-017-4050-6 (PMC5563062; doi:10.1186/s12864-017-4050-6)
Supplement: Supplementary file 1 — Correlation plot between genome proportion and total read count per million reads related to the 248 clusters annotated as LTR-REs (acronyms as in Table 1). (PDF 222 kb) [file 12864_2017_4050_MOESM1_ESM.pdf]

Figure S1.

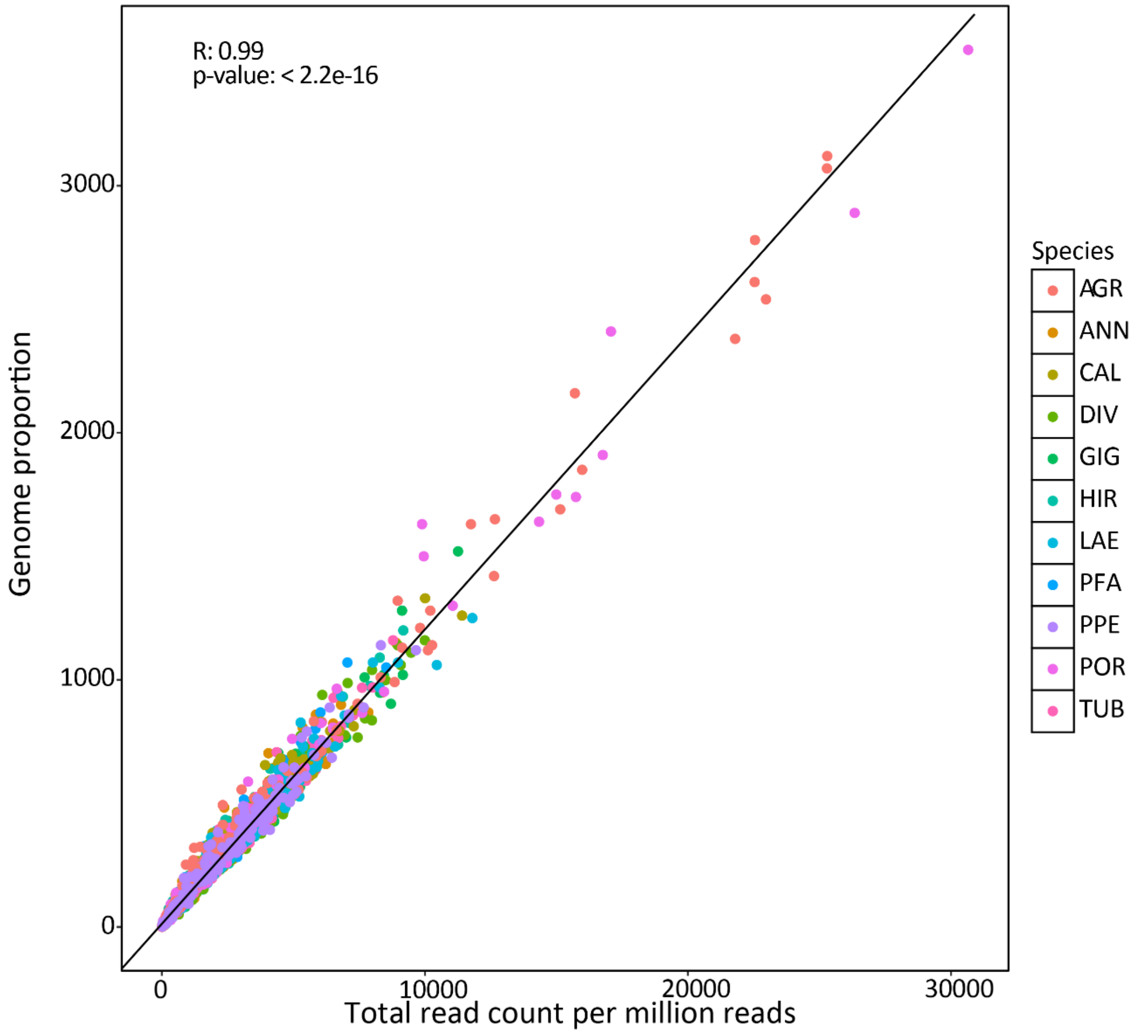

Correlation plot between genome proportion and total read count per million reads related to the 248 clusters annotated as LTR-REs (acronyms as in Table 1).
