# Supplementary material for: Genome-wide analysis of LTR-retrotransposon diversity and its impact on the evolution of the genus Helianthus (L.)
Source: BMC Genomics. 2017 Aug 18;18:634. doi: 10.1186/s12864-017-4050-6 (PMC5563062; doi:10.1186/s12864-017-4050-6)
Supplement: Supplementary file 2 — Distance tree of LTR-Copia RT domains of 10 species and one subspecies of Helianthus subjected to NJ analysis. Bootstrap values higher than 0.6 are shown with asterisk. Bar represents the nucleotide distance. Outgroups are RT domains of other species. Distance tree of LTR-Gypsy RT domains of 10 species and one subspecies of Helianthus subjected to NJ analysis. Bootstrap values higher than 0.6 are shown with asterisk. Bar represents the nucleotide distance. Outgroups are RT domains of other species. (PDF 487 kb) [file 12864_2017_4050_MOESM2_ESM.pdf]

Figure S2.

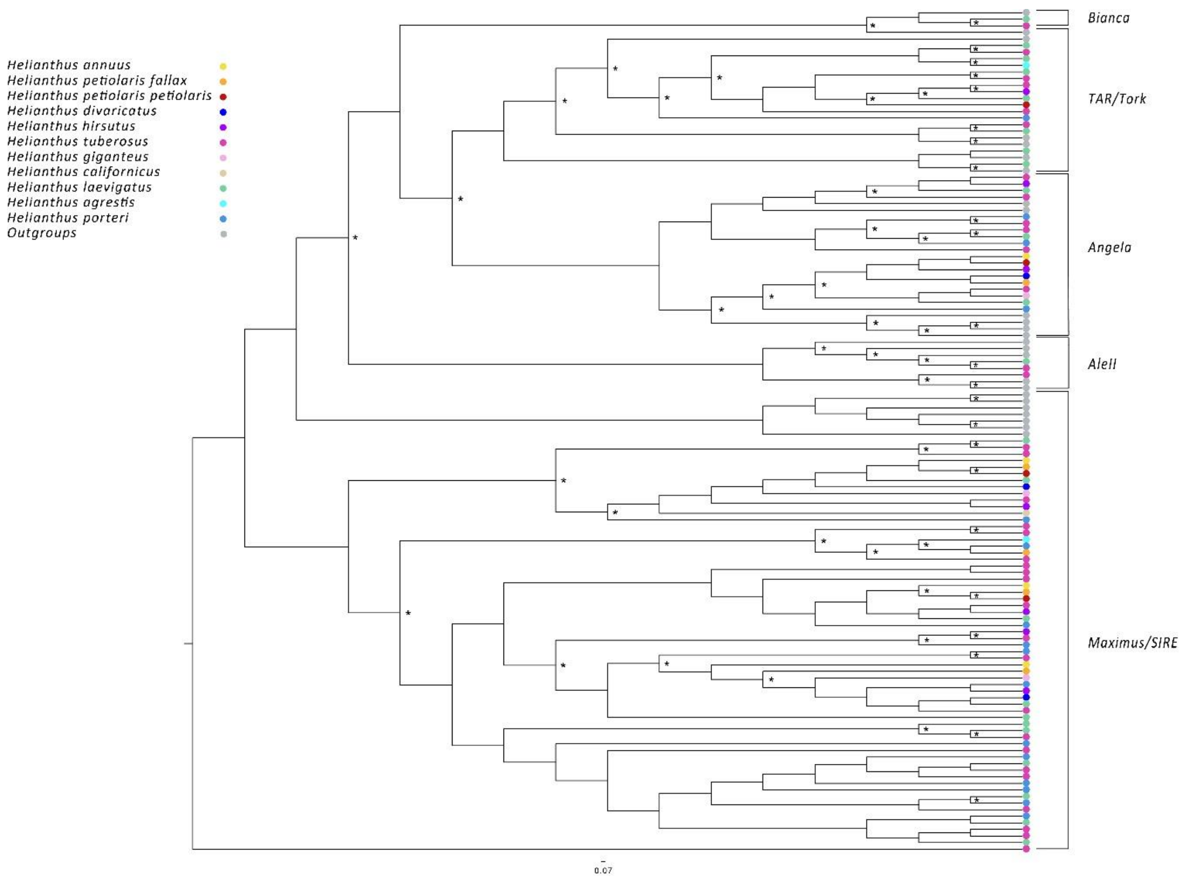

Distance tree of LTR-*Copia* RT domains of 10 species and one subspecies of *Helianthus* subjected to NJ analysis. Bootstrap values higher than 0.6 are shown with asterisk. Bar represents the nucleotide distance. Outgroups are RT domains of other species.

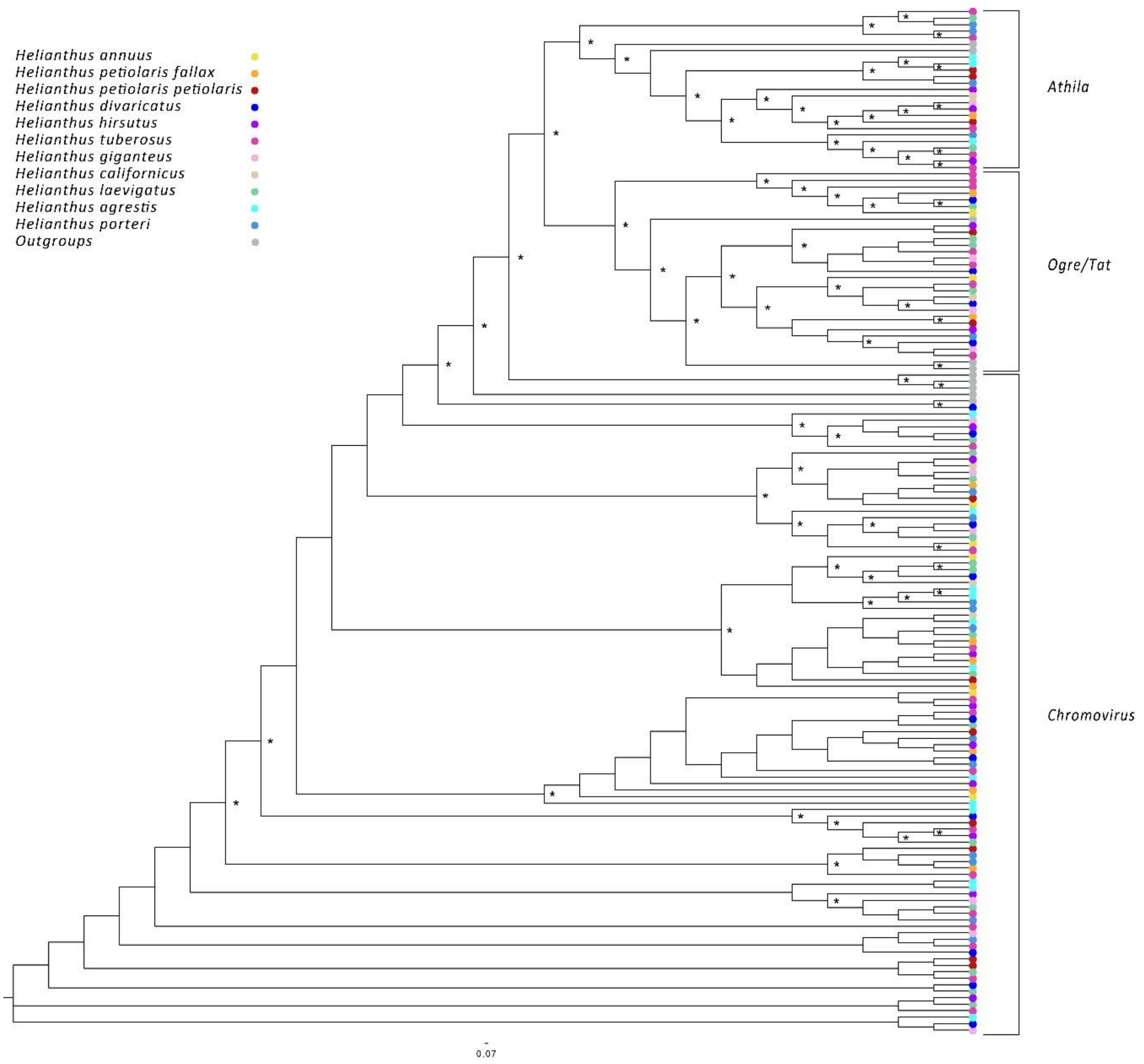

Distance tree of LTR-*Gypsy* RT domains of 10 species and one subspecies of *Helianthus* subjected to NJ analysis. Bootstrap values higher than 0.6 are shown with asterisk. Bar represents the nucleotide distance. Outgroups are RT domains of other species.
