# Supplementary material for: Genome-wide analysis of LTR-retrotransposon diversity and its impact on the evolution of the genus Helianthus (L.)
Source: BMC Genomics. 2017 Aug 18;18:634. doi: 10.1186/s12864-017-4050-6 (PMC5563062; doi:10.1186/s12864-017-4050-6)
Supplement: Supplementary file 3 — Sequence composition of LTR-Copia-RE-related clusters. The size of the rectangle is proportional to the genome proportion of a cluster for each species (acronyms as in Table 1). Bar plot in the top row shows the size of the clusters as number of reads in the comparative analysis. Upper lines label groups of clusters as asses by a hierarchical clustering of the results. The percentage of reads included in the group is shown in parentheses. The colour of the rectangles corresponds to the lineage of the Copia LTR-RE. All Copia-related repeats were shared among all the 10 species and one subspecies, with some peculiarities. In fact, differences were found even within lineages, producing 14 groups of RE sublineages with different abundance patterns, which accounted for 0.03 to 3.53% of the genome. For example, group 10, although being the most abundant (3.53% of the genome on the whole), was made up by nine clusters (eight annotated as Maximus/SIRE and one as Copia-unknown), that were represented in all species, but showed the largest abundance in H. porteri. On the contrary, the eight sublineages of group 12, annotated as Maximus as well, showed the highest genome proportions especially in H. agrestis and, to a lesser extent, in the Helianthus section. (PDF 454 kb) [file 12864_2017_4050_MOESM3_ESM.pdf]

Figure S3.

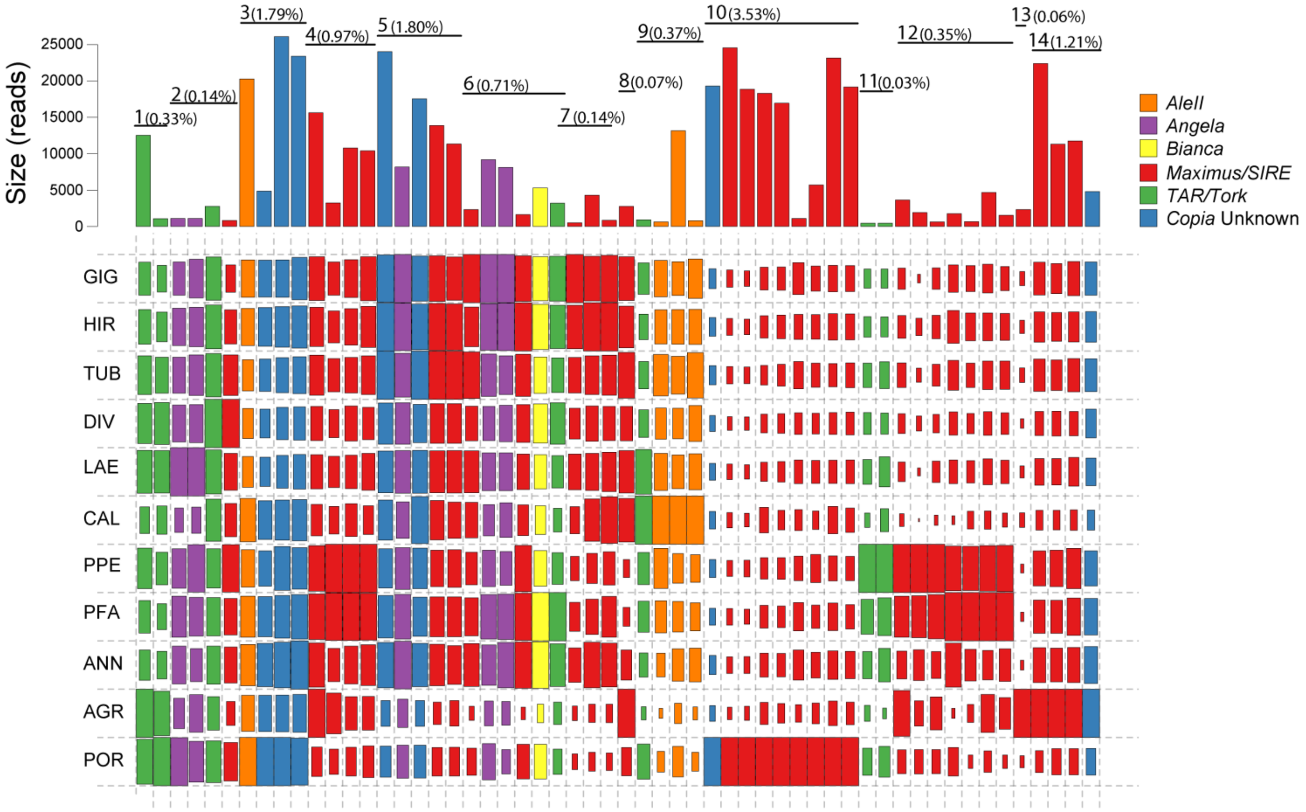

-Sequence composition of LTR-*Copia*-RE-related clusters. The size of the rectangle is proportional to the genome proportion of a cluster for each species (acronyms as in table 1). Bar plot in the top row shows the size of the clusters as number of reads in the comparative analysis. Upper lines label groups of clusters as asses by a hierarchical clustering of the results. The percentage of reads included in the group is shown in parentheses. The colour of the rectangles corresponds to the lineage of the *Copia* LTR-RE.

All *Copia*-related repeats were shared among all the 10 species and one subspecies, with some peculiarities. In fact, differences were found even within lineages, producing 14 groups of RE sublineages with different abundance patterns, which accounted for 0.03 to 3.53% of the genome. For example, group 10, although being the most abundant (3.53% of the genome on the whole), was made up by nine clusters (eight annotated as *Maximus/SIRE* and one as *Copia*-unknown), that were represented in all species, but showed the largest abundance in *H. porteri*. On the contrary, the eight sublineages of group 12, annotated as *Maximus* as well, showed the highest genome proportions especially in *H. agrestis* and, to a lesser extent, in the *Helianthus* section.
