# Supplementary material for: Genome-wide analysis of LTR-retrotransposon diversity and its impact on the evolution of the genus Helianthus (L.)
Source: BMC Genomics. 2017 Aug 18;18:634. doi: 10.1186/s12864-017-4050-6 (PMC5563062; doi:10.1186/s12864-017-4050-6)
Supplement: Supplementary file 5 — Distribution (on a logarithmic scale) of the ratio between the average coverage of 5′-LTR and respective coding (inter-LTR) DNA sequence related to 25 Copia (left) and 16 Gypsy (right) isolated REs, grouped per species. Species are distributed by increasing genome size keeping separated ploidy levels. Diploid species are in red, tetraploid in blue and hexaploid in green. The boxes represent the 25–75%, whiskers the whole range of values and dots the outliers. The lines in the boxes represent the medians of the distributions. Within diploid or tetraploid species, those indicated by different letters are significantly different (p < 0.05) according to Tukey’s test. (PDF 426 kb) [file 12864_2017_4050_MOESM5_ESM.pdf]

Figure S4.

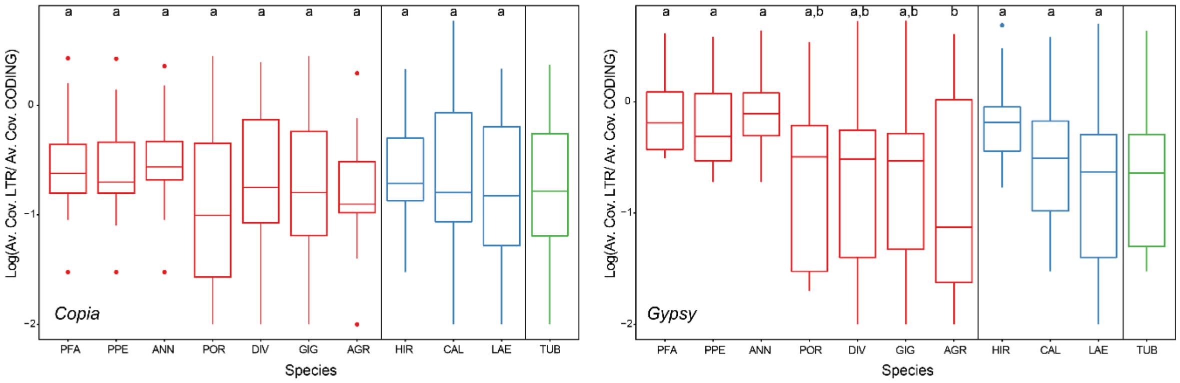

Distribution (on a logarithmic scale) of the ratio between the average coverage of 5′-LTR and respective coding (inter-LTR) DNA sequence related to 25 *Copia* (left) and 16 *Gypsy* (right) isolated REs, grouped per species. Species are distributed by increasing genome size keeping separated ploidy levels. Diploid species are in red, tetraploid in blue and hexaploid in green. The boxes represent the 25–75 %, whiskers the whole range of values and dots the outliers. The lines in the boxes represent the medians of the distributions. Within diploid or tetraploid species, those indicated by different letters are significantly different ($p < 0.05$) according to Tukey's test.

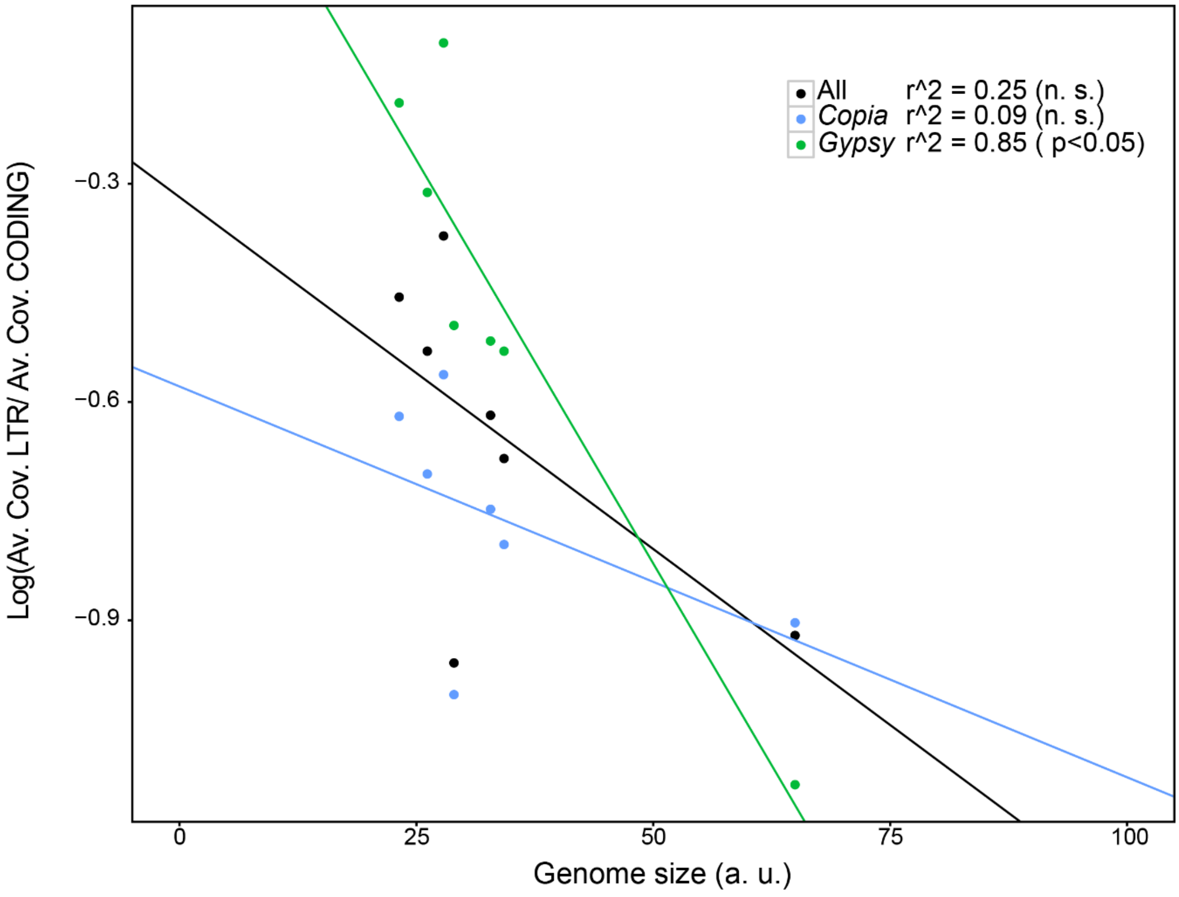

Correlation between genome size and LTR/coding region average coverage ratio in 6 diploid species and one subspecies of *Helianthus*. Correlation was calculated using the whole set of 41 intact retrotransposons (in black) and keeping separated 25 *Copia* (in blue) and 14 *Gypsy* (in green) REs. The correlation was significant for *Gypsy* REs (n.s. = not significant).
